# Comparison of electromagnetic navigation-guided and conventional blind nasojejunal tube placement in mechanically ventilated patients: a prospective non-randomized controlled study with two stages

**DOI:** 10.3389/fnut.2025.1674298

**Published:** 2026-01-20

**Authors:** You Yuan, Jing Fu, Benjing Wang, Mingli Zhong, Xi Luo, Yuqiang Wang, Qian Luo, Xia Zhang, Rujun Hu

**Affiliations:** 1 Department of Critical Care Medicine, Affiliated Hospital of Zunyi Medical University, Zunyi, Guizhou, China; 2 Department of Nursing, Affiliated Hospital of Zunyi Medical University, Zunyi, Guizhou, China; 3 School of Nursing, Zunyi Medical University, Zunyi, Guizhou, China; 4 Department of Critical Care Medicine, Zhejiang Provincial People’s Hospital Bijie Hospital, Bijie, Guizhou, China

**Keywords:** electromagnetic navigation, nasojejunal tube, mechanical ventilation, enteral nutrition, bedside procedure, blind insertion, clinical efficiency

## Abstract

**Background:**

During enteral nutrition support for mechanically ventilated patients, nasojejunal tube (NJT) placement encounters significant challenges in operational efficiency due to the lack of highly effective bedside techniques. Compared to the conventional blind insertion method for NJT placement (CBN-P), the electromagnetic navigation-guided placement technique (ENG-P) potentially offers superior advantages in terms of safety, procedural efficacy, cost-effectiveness, and the timeliness of clinical decision-making. However, the existing body of research in this area remains limited.

**Objective:**

The study aimed to evaluate and compare the clinical efficacy of ENG-P vs. CBN-P in mechanically ventilated patients.

**Methods:**

This was a prospective, non-randomized, two-phase cohort study. Patients requiring NJT placement were divided into control (CBN-P) and intervention (ENG-P) groups, enrolled from December 2024 to February 2025 and April to June 2025, respectively. Primary and secondary outcomes included first-attempt success rate, procedure duration, reinsertion frequency, patient discomfort, operator satisfaction, economic costs, clinical decision-making efficiency, and complication rates.

**Results:**

The ENG-P cohort demonstrated statistically superior performance compared to the CBN-P group, with significantly higher first-attempt success rates (81.36% vs. 65.85%, *P* = 0.042), shorter procedure duration (median 18 vs. 20 min, *P* < 0.001), and fewer re-insertion attempts (median 0 vs. 1, *P* = 0.001). No significant intergroup differences were found in patient discomfort, as measured by CPOT scores (*P* = 0.074), or in overall procedural success rates (84.75% vs. 78.05%, *P* = 0.253). The ENG-P technique showed notable improvements in several parameters: increased operator satisfaction (median score 8 vs. 6, *P* < 0.001), fewer radiographic confirmations required (*P* = 0.004), lower procedural costs (*P* = 0.005), and higher jejunal placement accuracy (81.36% vs. 40.24%, *P* < 0.001). In terms of clinical decision-making efficiency, the ENG-P group had a significantly shorter time from decision to enteral nutrition initiation (median 9.0 vs. 11.0 h, *P* = 0.001). However, no significant differences were observed in decision-to-placement time or decision-to-first radiographic confirmation time (both *P* > 0.05). Complication rates, including mucosal injury, coughing reflex, and tube obstruction, were similar between the two groups (all *P* > 0.05).

**Conclusion:**

ENG-P technique offers significant advantages over CBN-P, including higher first-attempt success rates, shorter procedure durations, fewer reinsertions, and lower healthcare costs. Additionally, it enables earlier enteral nutrition initiation while maintaining a favorable safety profile, making it the optimal choice for bedside NJT placement in ICU settings.

**Clinical trial registration:**

http://www.chictr.org.cn, identifier ChiCTR2500108091.

## 1 Introduction

In critically ill patients receiving mechanical ventilation within intensive care units (ICUs), endotracheal intubation routinely obstructs adequate oral nutritional intake, consequently mandating enteral nutrition delivery through nasogastric tubes (NGT) or nasojejunal tubes (NJT) (1, 2). Current clinical evidence corroborates that post-pyloric feeding utilizing NGJ tubes not only optimizes nutritional indices but also substantially curtails hospitalization periods, lowers healthcare costs, and diminishes the occurrence of ventilator-associated pneumonia, gastric retention, and mortality rates (3–8).

In this study, 29.08% of the enrolled patients had severe acute pancreatitis (SAP). Historically, the “pancreatic rest” theory dominated clinical practice for five decades, mandating NJT placement as the exclusive route for SAP patients while prohibiting NGT use. However, emerging evidence challenges this paradigm. Recent studies suggest that gastric feeding does not stimulate pancreatic secretion and may align better with physiological processes (9–12). NGT shows comparable safety to NJT while offering advantages in procedural simplicity and cost-effectiveness, supporting its recommendation as the first-line option for routine cases. Despite this, clinical guidelines continue to recommend NJT for high-risk scenarios, including mechanical ventilation, mild gastroparesis, pyloric edema/obstruction, and significant cyst compression—conditions strongly associated with increased aspiration risk (13). For patients requiring enteral nutrition for over 30 days, transitioning to percutaneous endoscopic gastrostomy/jejunostomy (PEG/J) is recommended to minimize complications associated with prolonged nasoenteric tube use (14). The primary clinical challenge in practice centers on the utilization of NJT for high-risk patient populations.

Notwithstanding its clinical necessity, the success rate of blind bedside insertion procedures remains suboptimal at merely 20–90%, which significantly contributes to the substantially lower adoption rate of NJT compared to NGT (15–18). This clinical limitation predominantly arises from the current lack of efficient and precise bedside techniques for post-pyloric tube placement (19).

Contemporary nasojejunal placement methodologies demonstrate several substantial limitations (20). The prevailing technique predominantly depends on blind manual insertion, subsequently requiring radiographic verification of tube positioning. This methodology poses notable technical difficulties, as initial unsuccessful attempts frequently necessitate repeated blind insertions—a procedure correlated with complications including nasopharyngeal and gastrointestinal mucosal trauma (21, 22). Furthermore, repeated radiographic imaging for tube placement verification not only exposes patients and healthcare personnel to cumulative radiation but also escalates healthcare expenditures (23, 24). Ultrasound-guided methodologies, albeit efficacious in mitigating healthcare expenditures, manifest several inherent limitations encompassing elevated technical requisites necessitating dual-operator synchronization, extended procedural durations, and an enduring dependency on radiographic verification for definitive positioning (25–27). Endoscopic or fluoroscopy-guided placement within interventional suites, while offering direct visualization, is circumscribed by substantial constraints such as specialized technical prerequisites, augmented costs, and compromised temporal efficiency (28). These impediments are particularly pronounced in intensive care unit environments where critically ill patients necessitate uninterrupted monitoring; multiple life-support apparatuses restrict patient mobility; and intrahospital transportation to endoscopy or interventional facilities significantly amplifies patient safety hazards (29, 30).

Contemporary methodologies for verifying post-pyloric tube placement are associated with substantial clinical limitations. Conventional approaches predominantly depend on auscultation subsequent to air insufflation, whereas more sophisticated techniques utilize pH monitoring or radiographic confirmation. Nevertheless, these verification methodologies share a fundamental deficiency (31–33). They function exclusively as post-placement detection instruments, implemented solely after the completion of tube insertion. This reactive paradigm is incapable of preventing malpositioning incidents, instead identifying complications (such as tube misplacement) subsequent to their occurrence (30).

In September 2020, the Nasogastric Tube Special Interest Group of BAPEN, a registered charitable organization, published a comprehensive position statement regarding nasogastric tube safety, issuing an imperative call to action: “Time to put patient safety first” (34). The document underscores that nasogastric tube placement constitutes a sophisticated, high-risk clinical intervention. There exists a critical, unaddressed demand for advanced bedside technologies that can either enhance or supersede conventional verification methodologies (including auscultation, pH testing, and radiographic confirmation) to enhance procedural safety and reliability.

The evolution in NJT placement represents a significant technological innovation. This procedure utilizes an endoscope integrated with micro-visualization technology to guide the NJT through the nasopharyngeal cavity, esophageal tract, gastric chamber, and pyloric sphincter into the duodenum. The real-time visualization system, facilitated by a microscopic camera at the distal extremity, enables precise navigation by continuously monitoring morphological alterations in the mucosal architecture of different anatomical zones. This landmark-based methodology allows for accurate determination of the tube’s position throughout the insertion trajectory (35–37).

Path visualization technology for nasoenteric tube placement. This innovative technique utilizes weak magnetic field sensing integrated with advanced computational algorithms to facilitate real-time three-dimensional trajectory mapping during nasoenteric intubation procedures. A magnetic locator, precisely positioned at the xiphisternal junction as the coordinate origin, establishes a comprehensive three-dimensional reference system (X, Y, Z axes). A miniature magnetic sensor embedded within the NJT’s guidewire continuously monitors and records its trajectory as it progresses through the anatomical pathway, including the nasopharynx, esophagus, stomach, pylorus, and duodenum (within the defined magnetic field domain). The system performs continuous computational analysis and generates real-time three-dimensional trajectory mapping, thereby providing precise visual confirmation of the tube’s anatomical progression (30, 38).

Given the demonstrated superiority of electromagnetic navigation in providing real-time visualization, this prospective non-randomized controlled study aims to comprehensively evaluate the clinical efficacy of Electromagnetic Navigation-Guided Placement (ENG-P) compared with Conventional Blind Nasogastric Tube Placement (CBN-P) in mechanically ventilated patients. All procedures were performed by experienced intensive care nurses following standardized protocols. The comparative analysis included the following outcome measures: first-attempt success rate, procedural efficiency including total operation time and number of required adjustments, patient discomfort level assessed using the Critical-Care Pain Observation Tool (CPOT), incidence of procedure-related complications, economic cost analysis, and clinical decision-making efficiency.

If ENG-P proves capable of overcoming the limitations of conventional blind insertion techniques—such as reliance on radiographic localization and higher complication risks—it could substantially reduce malpositioning, mucosal injury, and procedure duration, while also lowering healthcare costs. This would not only enhance procedural safety and patient comfort but also optimize enteral nutrition management for critically ill patients and provide a more reliable option for enteral access in mechanically ventilated patients.

## 2 Materials and methods

### 2.1 Study design

This was a single-center, prospective, two-phase, non-randomized controlled study. It compared ENG-P and CBN-P nasojejunal tubes in mechanically ventilated patients. The study was conducted under real-world clinical conditions. It followed quasi-experimental principles and adhered to the TREND (Transparent Reporting of Evaluations with Nonrandomized Designs) guidelines.

### 2.2 Study environment and population

This study was conducted in the ICUs of Affiliated Hospital of Zunyi Medical University, Guizhou Province, China. The department, with 31 years of progressive development, currently operates two specialized ICU wards comprising 75 critical care beds and 61 invasive mechanical ventilators. The study population was strictly limited to mechanically ventilated patients requiring NJT placement during their ICU hospitalization.

### 2.3 Participants

#### 2.3.1 Recruitment, screening, and enrollment

Upon ICU admission, the research team provided eligible patients and their families with an overview of the study to obtain preliminary consent. For interested candidates, formal recruitment was initiated, explaining the study’s objectives, risks, and potential benefits to the patient or their legally authorized representative. Participants were screened according to predefined criteria. Those who did not meet the requirements were documented with specific exclusion reasons. Final enrollment was limited to patients who fully understood the study protocol, voluntarily provided written informed consent and met all selection criteria. The recruitment process adhered to the Declaration of Helsinki, was monitored by the Institutional Review Board, and maintained thorough documentation with ongoing privacy protections.

#### 2.3.2 Inclusion criteria

(1)    Admitted to ICU; Age ≥ 18; Mechanically ventilated patients.
(2)    Clinically indicated for post-pyloric enteral nutrition via NJT.
(3)    At least one chest radiograph available for tube position confirmation.
(4)    Informed consent signed by the patient or legally authorized representative.

#### 2.3.3 Exclusion criteria

(1)    Patients not receiving mechanical ventilation; individuals with nasal trauma or anatomical abnormalities that contraindicate transnasal intubation.
(2)    Patients exhibiting any of the following conditions: Active upper gastrointestinal hemorrhage; gastrointestinal perforation or fistula; intestinal necrosis or obstruction; esophageal or gastric varices or strictures; post-surgical alterations in gastrointestinal anatomy; severe coagulopathy (INR > 3.0 or platelet count < 50 × 10^9^/L).
(3)    X-ray positioning is not acceptable for patients, such as pregnant women.
(4)    Patients or their legally authorized representatives who decline participation; absence of signed informed consent for NJT placement.

#### 2.3.4 Sample size

The primary endpoint of this clinical investigation was the procedural success rate, operationally defined as radiographically confirmed accurate NJT placement. Through an exhaustive literature review, we projected that the Control group (employing blind insertion technique) would demonstrate a 70% success rate (*p*0 = 0.70), whereas the Experimental group (utilizing electromagnetic navigation technology) would achieve a 91.4% success rate (*p*1 = 0.914) (3, 9, 18). The two-tailed significance level was set at α = 0.05, and the statistical power (1 −β) was 80%. Based on formula 1, the sample size calculation indicated that at least 50 patients were required per group. Considering a potential 10% attrition rate, the actual sample size per group was adjusted to approximately 55 patients, resulting in a total sample size of 110 patients.


$$n=\frac{{\left(Z_{a/2}+Z_{\beta }\right)}^{2}\times \left(p_{1}\,\left(1-p_{1}\right)+p_{0}\,\left(1-p_{0}\right)\right)}{{\left(p_{1}-p_{0}\right)}^{2}}$$ (1)

### 2.4 Trial equipment

Both groups used the same model of NJT with specifications of an internal diameter of 3 mm, an external diameter of 4 mm, and a length of 1,450 mm, for post-pyloric enteral nutrition support. Stethoscopes were used to confirm the position of the nasoenteric tube, and 50 ml syringes were used for aspirating or injecting gas.

**FIGURE 1:**
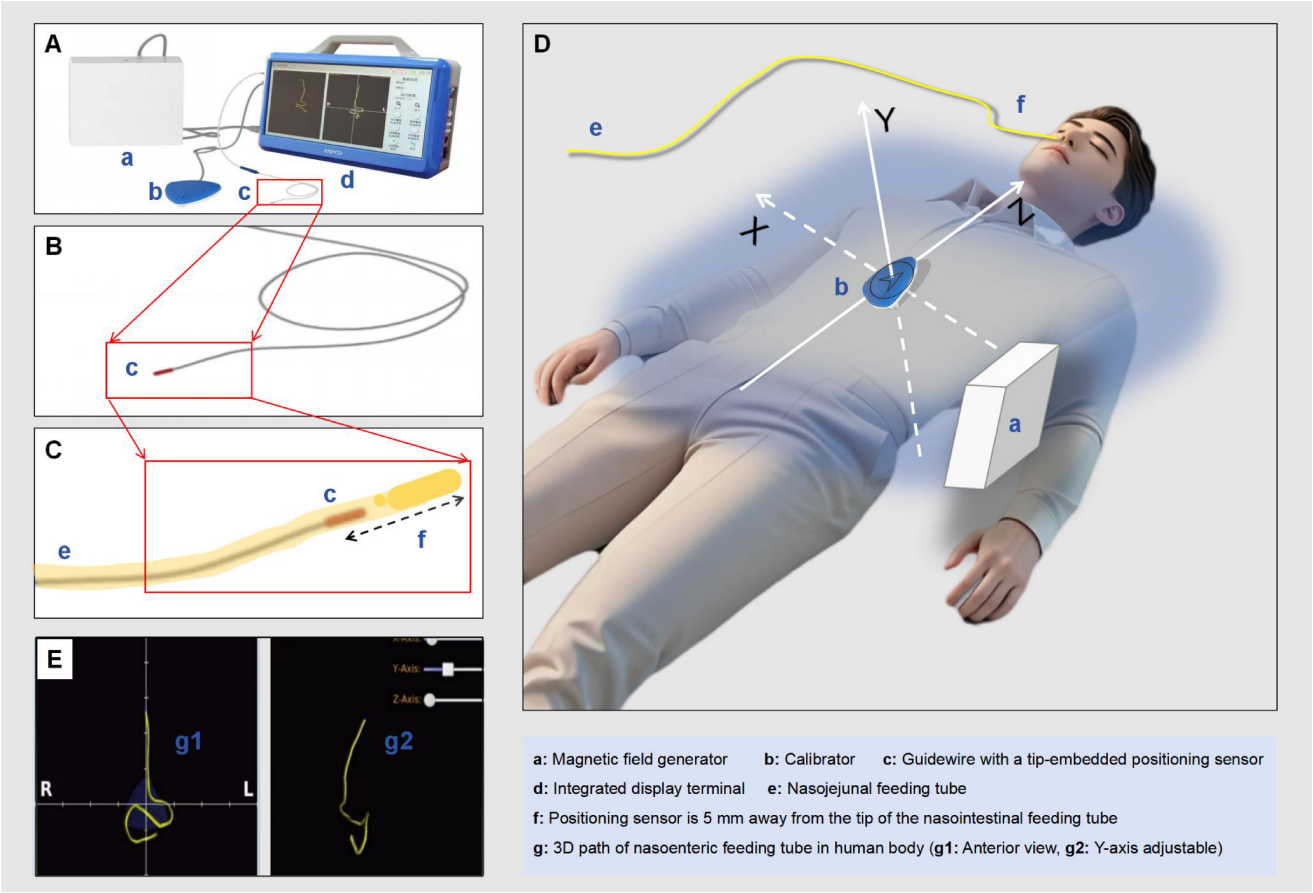
Principles of electromagnetic navigation-guided placement. **(A)** Magnetic field generator; **(B)** Calibrator; **(C)** Guidewire with a tip-embedded positioning sensor; **(D)** Integrated display terminal; **(E)** Nasojejunal feeding tube; **(F)** The positioning sensor is shown 10 mm away from the tip of the nasointestinal feeding tube; **(G)** The 3D path of the nasoenteric feeding tube inside the human body is depicted; (g1) anterior view and (g2) Y-axis adjustable view are clearly indicated.

The electromagnetic navigation system for nasoenteric tube placement comprises four fundamental components (Figure 1A): (a) a magnetic field generator, (b) a calibration device, (c) a guidewire incorporating a tip-embedded positioning sensor, and (d) an integrated display terminal. The guidewire (c) exhibits a length of 1,445 mm with a diameter less than 1.5 mm (Figure 1B). Preceding intubation, the magnetic field generator (a) is strategically positioned adjacent to the patient’s thoracic region, generating a low-intensity dynamic electromagnetic field extending from the nasal cavity to the pelvic area. This electromagnetic field constitutes the fundamental framework for spatial localization and orientation computations performed by both the guidewire (c) and calibration device (b). The calibration device (b), equipped with miniature sensor coils, is precisely positioned at the xiphisternal junction. This apparatus serves a dual function: detecting electromagnetic field signals for autonomous localization and establishing a three-dimensional Cartesian coordinate system (X, Y, Z) with itself as the reference origin (Figure 1D). During the intubation procedure, the sensor-embedded guidewire (c) is inserted into the nasoenteric tube, maintaining its tip position 5 mm proximal to the tube’s distal extremity (Figure 1C). The guidewire’s integrated sensor coils facilitate continuous electromagnetic field detection, enabling real-time monitoring of the tube’s trajectory. The integrated display terminal (d) executes advanced signal processing algorithms and image reconstruction, transforming real-time positional data acquired from both the calibration device and guidewire sensors into dynamic three-dimensional graphical representations (Figure 1E).

The electromagnetic field generated by the system is low in intensity and interacts only with the guidewire’s magnetic sensor. According to the device manual and clinical experience, within the normal range of use, the field does not interfere with other medical devices, including ECG monitors or pacemakers, nor is it affected by bedside equipment such as hemodialysis machines or ECMO devices. In addition, the device has passed electromagnetic compatibility (EMC) certification by the China National Medical Products Administration (NMPA), confirming its safety for clinical use.

### 2.5 Study interventions

#### 2.5.1 Patients grouping

This study adopts a two-phase, prospective cohort design with non-randomized, non-blinded allocation. The control group will be recruited from December 2024 to February 2025, followed by the experimental group from April to June 2025. Each cohort will comprise a minimum of 55 participants. All procedures will be conducted by certified nurses who have undergone specialized training and possess extensive catheterization experience.

#### 2.5.2 Control group: conventional blind nasogastric tube placement

The procedure for NJT placement necessitates the preparation of essential materials, such as a NJT, syringes, gauze, and liquid paraffin. The blind insertion technique integrates gastric insufflation with positional adjustments. Initially, the tube is inserted into the stomach using standard nasogastric intubation techniques, and its placement is confirmed. Subsequently, a limited volume of air (<300 mL, not exceeding 500 mL) is insufflated into the stomach, and the patient is positioned in the right lateral decubitus position to facilitate the gradual advancement of the tube into the jejunum. Preliminary confirmation of correct tube positioning involves three complementary methods: auscultation to detect the progression of maximal air-entry sounds from the xiphoid process to the right and then left upper abdominal quadrants; the vacuum test, characterized by smooth injection of water but limited aspiration with less than one-third of the injected volume returned; and pH testing, where a jejunal placement is indicated by an aspirate pH greater than 7.0. After confirming the position, the guidewire is removed, and the tube is secured. Final confirmation is obtained through abdominal radiography. In the event of gastric coiling, partial withdrawal followed by reinsertion of the tube may be performed. Throughout the procedure, continuous monitoring for adverse events is conducted, and all procedural data are systematically documented.

#### 2.5.3 Experimental group: electromagnetic navigation-guided placement

Prepare the electromagnetic navigation system. Position the patient in a supine or semi-recumbent posture, ensuring clear identification of the xiphoid process. Secure the calibration device and the magnetic field generator. Connect the distal end of the metal guidewire, which is equipped with an electromagnetic sensor, to the integrated display terminal, and then insert it into the NJT to initiate the tube placement procedure. Insert the feeding tube through one of the patient’s nostrils. Because the tube contains the metal guidewire, the patient’s gastrointestinal tract and the tube’s position can be clearly visualized on the integrated display terminal under electromagnetic guidance. Once the tube reaches the stomach, gas (not exceeding 300 mL, with an upper limit of 500 mL) may be selectively introduced into the stomach. Subsequently, advance the tube slowly at a rate of 1–2 cm per push after placing the patient in the right lateral position. Resistance is commonly encountered when the pylorus is reached; at this point, slightly withdraw the guidewire and then slowly advance it again until the tube passes the Treitz ligament. The position of the NJT can be further verified by adjusting the 3D path view.

During tube placement, the ENG-P system automatically generates three-dimensional images of the tube trajectory. However, if the tube remains at the same gastrointestinal location and moves slightly, repeated images may occur, producing artifacts and locally reduced image quality. In such cases, the operator can select “Stop Drawing” on the display to automatically clear existing images; subsequently, selecting “Start Drawing” and withdrawing the guidewire will generate a high-quality three-dimensional image of the nasoenteric tube trajectory. Alternatively, the guidewire can be retracted to the nasal cavity while the tube remains in place, reinserted along the tube, and “Start Drawing” selected again to generate a new complete high-quality three-dimensional image. This adjustment process is simple, does not require complex system reconfiguration, and trained clinical staff can easily perform it.

After removing the guidewire, secure the tube appropriately. Finally, confirm the position of the tube’s tip using X-ray imaging. If the positioning is inaccurate, retract the tube into the stomach and repeat the procedure. Throughout the placement process, adhere to the same protocol as the control group regarding precautions, monitoring and management of potential complications, and data recording.

### 2.6 Observational indicators and measurements

#### 2.6.1 Primary outcomes and secondary outcomes

The primary outcome is the first-attempt success rate, defined as the proportion of patients in whom the NJT is successfully placed in the jejunum during the initial attempt. The secondary outcome is the overall placement success rate, which encompasses all attempts and reflects the proportion of patients who ultimately achieve successful NJT placement. The time required for the first placement is measured from the moment the tube is inserted into the nostril until the completion of the procedure. Pain during the procedure is evaluated using the which assesses four behavioral indicators: facial expression, body movements, muscle tension, and vocal response. Each indicator is scored from 0 to 2, resulting in a total score ranging from 0 to 8, corresponding to the following pain levels: no pain (0), mild pain (1–3), moderate pain (4–6), and severe pain (7–8) (40, 41). Additional tube adjustments refer to the total number of subsequent manipulations required to achieve successful placement. If successful placement is not achieved within 20 min, withdrawing the tube into the stomach for reinsertion or allowing another operator to perform the procedure is considered one additional adjustment, reflecting both procedural difficulty and the workload on medical personnel. The operator’s prediction of successful jejunal placement after the first attempt is classified into three categories: successful placement, unsuccessful placement, or uncertain. Operator satisfaction with the initial placement is rated on a numerical scale from 0 to 10, where 0 indicates the lowest level of satisfaction and 10 indicates the highest. Finally, the cost of the entire placement procedure, including both material and operational expenses, is recorded to evaluate its economic impact.

#### 2.6.2 Timeliness of clinical decision-making indicators

The time from decision to catheter placement (TDCP) refers to the interval between the physician’s decision to perform tube placement and the actual start of the procedure. The time from decision to first X-ray confirmation (TDFXC) refers to the interval between the physician’s decision to perform tube placement and the first radiographic examination. The time from decision to initiation of enteral nutrition (TDIEN) refers to the interval between the physician’s decision to perform tube placement and the commencement of enteral feeding in the patient.

#### 2.6.3 Adverse events

Placement-related complications include mucosal injury, coughing, and tube misplacement, as well as issues arising during tube maintenance, such as displacement, blockage, and aspiration. The frequency of these complications is recorded based on their occurrence.

### 2.7 Quality control and data analysis

#### 2.7.1 Quality control

Before enrollment, baseline data were collected, including age, gender, Body Mass Index (BMI) scores, major clinical diagnoses, Richmond Agitation-Sedation Scale (RASS) scores, CPOT scores, Acute Gastrointestinal Injury (AGI) scores, the use of prokinetic agents, and gastrointestinal decompression. This information was used to comprehensively characterize the patient population and serve as a foundation for intergroup comparisons. Additionally, strict quality control measures were implemented throughout the entire tube placement procedure, covering aspects such as personnel qualification verification, standardized operational protocols, real-time monitoring, complication management, and accurate data recording and entry. These measures were designed to minimize potential biases introduced by operators and evaluators.

#### 2.7.2 Data analysis

Data analysis was conducted using SPSS version 29.0 (IBM, Armonk, NY, United States) and R version 4.3.3 (2024-02-29), in conjunction with the Storm Statistical Platform.^1^ The sample characteristics were summarized using frequencies, percentages, means, standard deviations (SD), medians, and interquartile ranges (IQR). Group comparisons were performed using one-way ANOVA for normally distributed data or non-parametric tests for non-normally distributed data. Categorical variables were analyzed using the chi-square test or Fisher’s exact test, as appropriate. All statistical tests were two-tailed, and a *p* < 0.05 was considered statistically significant.

## 3 Results

### 3.1 Recruitment and dropout

A total of 166 participants were initially screened and enrolled in the study. Following the application of the inclusion and exclusion criteria, 146 participants were ultimately included and divided into two phases: the control group (CBN-P), comprising 87 patients enrolled from December 2021 to February 2025, and the experimental group (ENG-P), consisting of 59 patients enrolled from April to June 2025.

During the study, five patients in the CBN-P group underwent tube placement outside of the ICU, with four patients receiving this procedure via interventional radiography and one patient undergoing it through endoscopy. In contrast, no patients in the ENG-P group had the procedure performed outside of the ICU. Ultimately, a total of 81 patients received CBN-P treatment, while 59 patients were treated with ENG-P.

In the CBN-P group, 54 patients successfully underwent first-attempt tube placement.

Following additional adjustments, the number of patients with confirmed tube placement via X-ray increased to 64. 9 patients experienced placement failure, and an additional 9 did not undergo X-ray confirmation for their placements; among these, 4 required re-placement outside the ICU. In the ENG-P group, 48 patients achieved successful first-attempt tube placement, which was subsequently confirmed by X-ray in 50 cases after further adjustments were made. 2 patients encountered placement failure, while 7 did not have an X-ray to verify tube position; 1 of these required re-placement outside the ICU.

**FIGURE 2:**
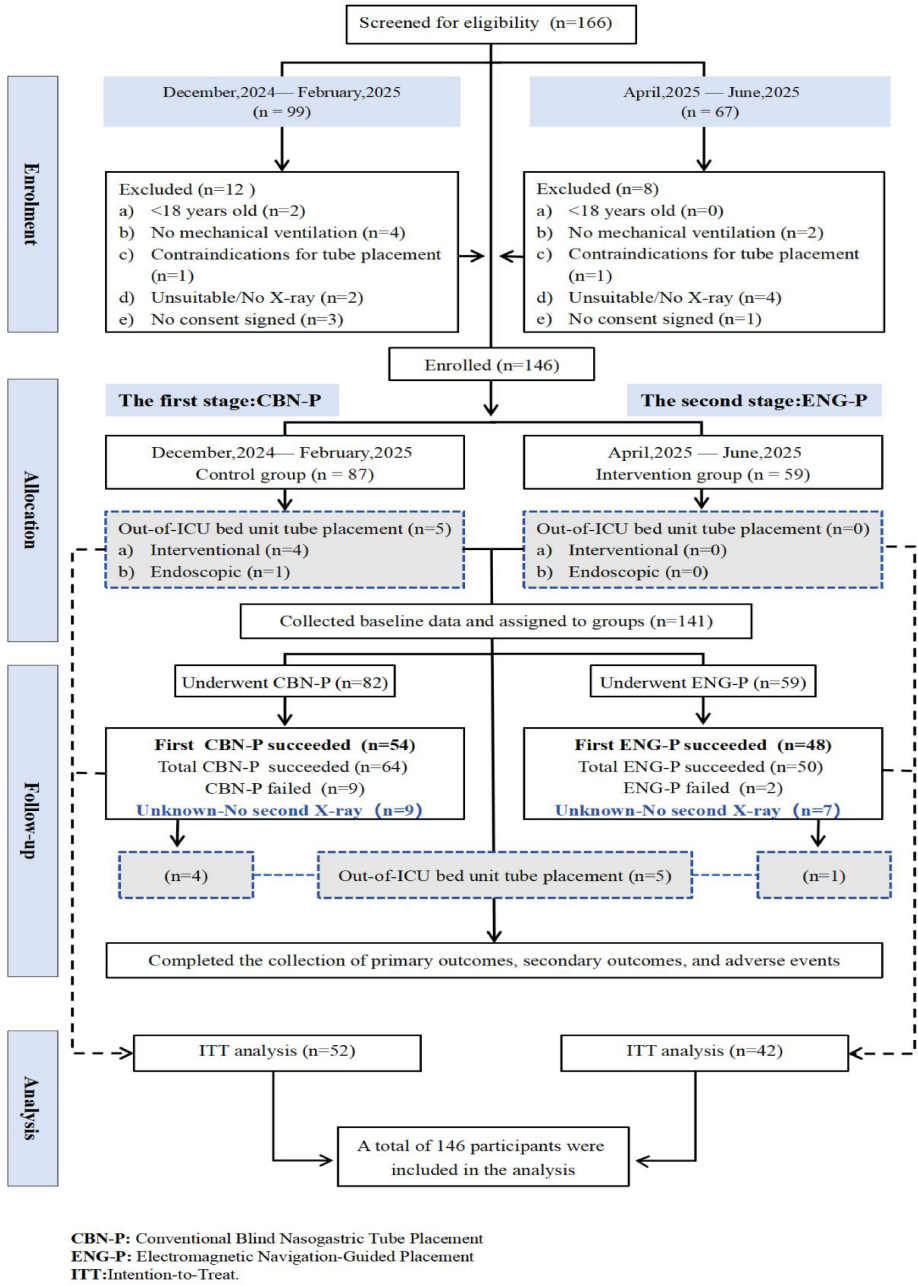
The patients study participation pathway.

Baseline data as well as primary and secondary outcomes were meticulously documented alongside adverse events, and intention-to-treat (ITT) analysis was conducted. The patient participation process is illustrated in Figure 2.

### 3.2 Baseline characteristics

Comparative analysis of patient characteristics between the two groups, encompassing age, gender, BMI scores, primary clinical diagnoses, RASS scores, CPOT scores, AGI scores, utilization of prokinetic agents, and gastrointestinal decompression procedures revealed no statistically significant differences. This finding suggests that the groups are comparable in these aspects. Detailed results are presented in Table 1.

**TABLE 1:** Comparison of basic data.

Variables	Total (*n* = 141)	CBN-P (*n* = 24)	ENG-P (*n* = 25)	Statistical value	*P*-value
Age, mean ± SD	60.84 ± 16.09	59.91 ± 15.99	62.12 ± 16.29	*t* = −0.80	0.424
Gender, n(%)				χ^2^ = 0.02	0.880
Male	85 (60.28)	49 (59.76)	36 (61.02)		
Female	56 (39.72)	33 (40.24)	23 (38.98)		
Body mass index, M (Q_1_, Q_3_)	21.20 (19.20, 23.30)	21.26 (18.99, 23.55)	20.63 (19.70, 23.22)	*Z* = −0.37	0.711
APACHE-II, M (Q_1_, Q_1_)	25.00 (22.00, 28.00)	25.00 (21.25, 30.75)	25.00 (23.00, 28.00)	Z = −0.81	0.416
Clinical diagnoses,n(%)				χ^2^ = 7.50	0.186
Severe pneumonia	40 (28.37)	22 (26.83)	18 (30.51)		
Severe acute pancreatitis	41 (29.08)	27 (32.93)	14 (23.73)		
Traumatic brain injury	28 (19.86)	14 (17.07)	14 (23.73)		
Sepsis	12 (8.51)	4 (4.88)	8 (13.56)		
Polytrauma	10 (7.09)	7 (8.54)	3 (5.08)		
Other	10 (7.09)	8 (9.76)	2 (3.39)		
RASS, n(%)				–	0.177
0	7 (4.96)	4 (4.88)	3 (5.08)		
−1	11 (7.80)	6 (7.32)	5 (8.47)		
−2	35 (24.82)	15 (18.29)	20 (33.90)		
−3	37 (26.24)	24 (29.27)	13 (22.03)		
−4	22 (15.60)	17 (20.73)	5 (8.47)		
−5	29 (20.57)	16 (19.51)	13 (22.03)		
CPOT score, n(%)				–	0.193
0	61 (43.26)	38 (46.34)	23 (38.98)		
1	52 (36.88)	25 (30.49)	27 (45.76)		
2	25 (17.73)	16 (19.51)	9 (15.25)		
3	3 (2.13)	3 (3.66)	0 (0.00)		
Acute gastrointestinal injury, n(%)				χ^2^ = 0.78	0.377
Grade I	132 (93.62)	75 (91.46)	57 (96.61)		
Grade II	9 (6.38)	7 (8.54)	2 (3.39)		
Prokinetic drug use, n(%)				χ^2^ = 0.15	0.700
Yes	50 (35.46)	28 (34.15)	22 (37.29)		
No	91 (64.54)	54 (65.85)	37 (62.71)		
Decompression status, n(%)				χ^2^ = 0.04	0.843
Yes	85 (60.28)	50 (60.98)	35 (59.32)		
No	56 (39.72)	32 (39.02)	24 (40.68)		

t, *t*-test; Z, Mann-Whitney test; χ^2^, Chi-square test; -, Fisher exact; SD, standard deviation; M, Median; Q1, 1st Quartile; Q3, 3st Quartile; CBN-P, Conventional Blind Nasogastric Tube Placement; ENG-P, Electromagnetic Navigation-Guided Placement.

### 3.3 Primary and Secondary outcomes

A comparative analysis of primary and secondary outcomes between the CBN-P and the ENG-P revealed superior performance of the ENG-P group across multiple key parameters. The first-attempt success rate demonstrated a statistically significant advantage in the ENG-P group compared to the CBN-P group (81.36% vs. 65.85%, *P* = 0.042), while the overall success rate showed no significant intergroup difference (84.75% vs. 78.05%, *P* = 0.253). Procedural duration was significantly reduced in the ENG-P group (median 18 min vs. 20 min, *P* < 0.001). Although no significant difference was observed in pain assessment (CPOT score) between the groups (*P* = 0.074), the ENG-P group exhibited superior procedural satisfaction (median 8 vs. 6, *P* < 0.001) and significantly fewer tube reinsertions (median 0 vs. 1, *P* = 0.001). Additionally, the ENG-P group demonstrated reduced radiographic examinations (*P* = 0.004) and lower associated costs (*P* = 0.005). Regarding jejunal placement prediction, the ENG-P group achieved a markedly higher success rate compared to the CBN-P group (81.36% vs. 40.24%, *P* < 0.001), as detailed in Table 2.

**TABLE 2:** Primary and secondary outcomes.

Variables	Total (*n* = 141)	CBN-P (*n* = 82)	ENG-P (*n* = 59)	Statistic	*P*
First successed, n(%)				χ^2^ = 4.12	0.042
No	39 (27.66)	28 (34.15)	11 (18.64)		
Yes	102 (72.34)	54 (65.85)	48 (81.36)		
Total successed, n(%)				χ^2^ = 2.75	0.253
No	11 (7.80)	9 (10.98)	2 (3.39)		
Yes	114 (80.85)	64 (78.05)	50 (84.75)		
Unknown-no second X-ray	16 (11.35)	9 (10.98)	7 (11.86)		
Procedure time, M (Q1, Q3)	20.00 (15.00, 24.00)	20.00 (16.00, 25.00)	18.00 (14.00, 22.00)	*Z* = −3.29	< 0.001
Procedure CPOT, M (Q1, Q3)	2.00 (1.00, 3.00)	3.00 (1.25, 3.00)	2.00 (1.00, 3.00)	*Z* = −1.79	0.074
Procedure satisfaction, M (Q1, Q3)	7.00 (6.00, 8.00)	6.00 (5.00, 8.00)	8.00 (6.00, 8.00)	*Z* = −4.11	< 0.001
Re-insertion attempts, M (Q1, Q3)	0.00 (0.00, 1.00)	1.00 (0.00, 2.00)	0.00 (0.00, 1.00)	*Z* = −3.22	0.001
X-ray times, M (Q1, Q3)	1.00 (1.00, 1.00)	1.00 (1.00, 1.75)	1.00 (1.00, 1.00)	*Z* = −2.87	0.004
COST, M (Q1, Q3)	886.00(886.00, 886.00)	886.00(886.00, 952.00)	886.00(886.00, 886.00)	*Z* = −2.84	0.005
Jejunal reach prediction, n(%)				χ^2^ = 27.87	< 0.001
Yes	81 (57.45)	33 (40.24)	48 (81.36)		
No	23 (16.31)	15 (18.29)	8 (13.56)		
Uncertain	37 (26.24)	34 (41.46)	3 (5.08)		

Z, Mann-Whitney test; χ^2^, Chi-square test; M, Median; Q1, 1st Quartile; Q3, 3st Quartile; CBN-P, Conventional Blind Nasogastric Tube Placement; ENG-P, Electromagnetic Navigation-Guided Placement.

### 3.4 Clinical decision-making timeliness

Regarding clinical decision timeliness, the ENG-P group demonstrated a statistically significant advantage over the CBN-P group in the time interval from decision to initiation of enteral nutrition (TDIEN), with a median duration of 9.0 h vs. 11.0 h in the CBN-P group (*P* = 0.001). Nevertheless, no statistically significant differences were observed between the groups for the time from decision to tube placement (TDCP) and time from decision to first radiographic confirmation (TDFXC). The detailed comparative data are presented as follows: TDCP (median 3.0 h in the ENG-P group compared to 2.5 h in the CBN-P group, *P* = 0.75) and TDFXC (median 4.0 h in the ENG-P group versus 5.0 h in the CBN-P group, *P* = 0.185), as illustrated in Table 3.

**TABLE 3:** Results of clinical decision-making time.

Variables (group)	Clinical decision-making timeliness		
	TDCP (time from decision to catheter placement)	TDFXC (time from decision to first X-ray confirmation)	TDIEN (time from decision to initiation of enteral nutrition)
Total (*n* = 141), M (Q1, Q3)	3.00 (1.00, 5.00)	5.00 (3.00, 7.00)	10.00 (7.00, 15.00)
CBN-P (*n* = 82), M (Q1, Q3)	2.50 (1.00, 4.00)	5.00 (3.00, 7.00)	11.00 (8.00, 16.00)
ENG-P (*n* = 59), M (Q1, Q3)	3.00 (1.00, 6.00)	4.00 (2.00, 7.00)	9.00 (5.00, 12.50)
Statistic	*Z* = −0.32	*Z* = −1.32	*Z* = −3.22
*P*	0.75	0.185	0.001

Z, Mann-Whitney test, M, Median, Q1, 1st Quartile, Q3, 3st Quartile; CBN-P, Conventional Blind Nasogastric Tube Placement; ENG-P, Electromagnetic Navigation-Guided Placement.

### 3.5 Adverse events

The incidence rates of mucosal injury bleeding and Choking cough were recorded at 5.08 and 8.47%, respectively, in the ENG-P cohort, both marginally lower than those observed in the CBN-P group (8.54% and 9.76%). In terms of maintenance-related adverse events, the ENG-P group exhibited incidence rates of catheter dislodgement, obstruction, and aspiration at 3.39, 3.39, and 1.69%, respectively, in comparison to 4.88, 4.88, and 2.44% in the CBN-P group, with no statistically significant intergroup differences observed. Notably, neither cohort reported any instances of catheter malposition, as detailed in Table 4.

**TABLE 4:** Adverse events.

Variables (group)	Procedure-related adverse events			Maintenance-related adverse events		
	Mucosal injury bleeding	Choking cough	Catheter malposition	Catheter dislodgement	Catheter blockage	Reflux aspiration
CBN-P (*n* = 82)	7 (8.54)	8 (9.76)	/	4 (4.88)	4 (4.88)	2 (2.44)
ENG-P (*n* = 59)	3 (5.08)	5 (8.47)	/	2 (3.39)	2 (3.39)	1 (1.69)
χ^2^	0.21	0.07	/	0.000	0.000	0.000
*P*	0.649	0.795	/	0.993	0.993	1.000

χ^2^, Chi-square test; CBN-P, Conventional Blind Nasogastric Tube Placement; ENG-P, Electromagnetic Navigation-Guided Placement.

### 3.6 Adjusted overall success rate and subgroup analyses

**FIGURE 3:**
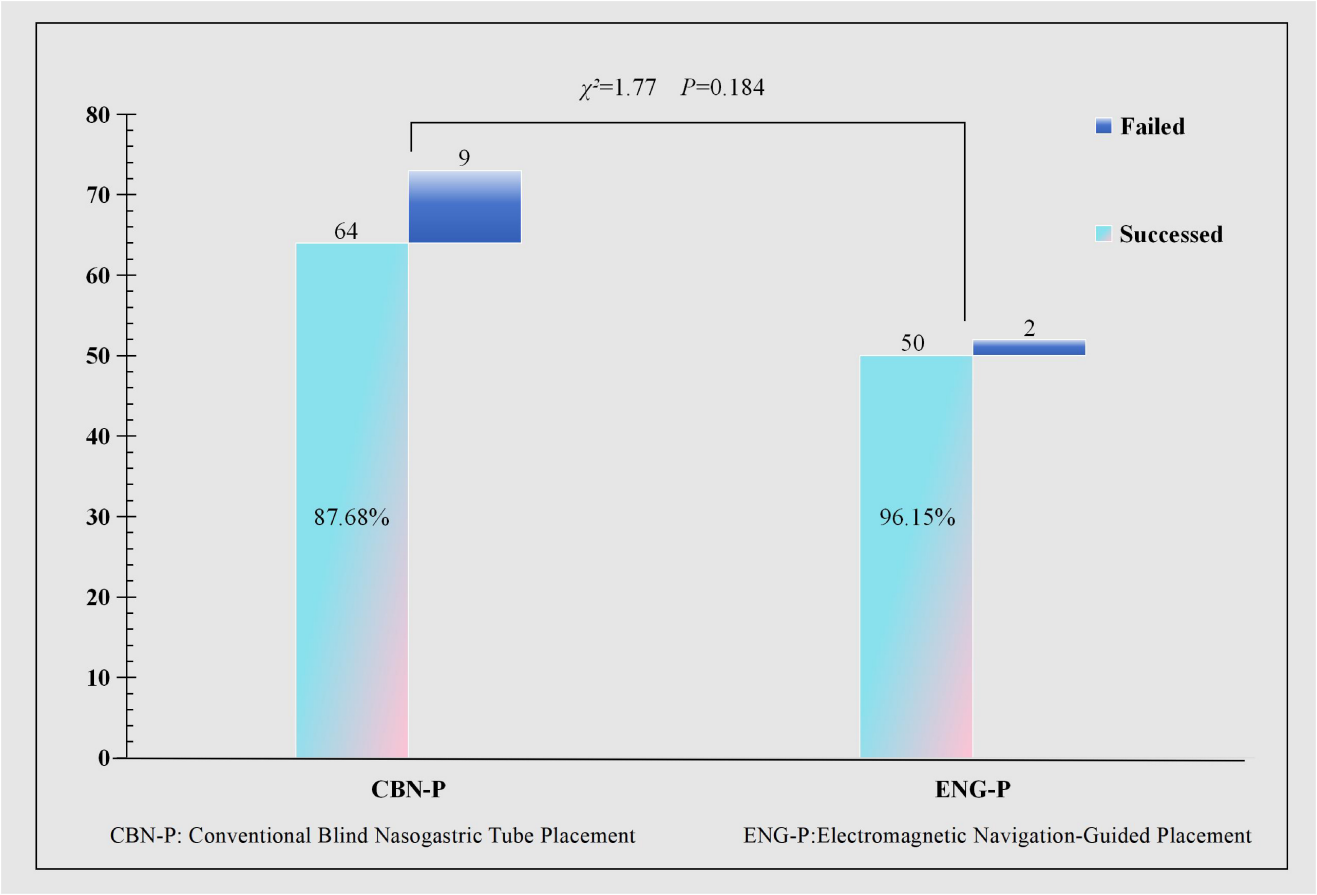
Calibrated final tube placement success rate.

Cases without secondary radiographic verification of nasojejunal tube (NJT) positioning were excluded from the analysis. After statistical adjustment, the overall success rates were 87.7% (64/73) in the CBN-P group and 96.2% (50/52) in the ENG-P group, with no significant difference between the two groups, as shown in Figure 3.

Within the ENG-P group, subgroup analyses were conducted based on sedation depth, divided into six subgroups according to RASS scores, and the use of gastrointestinal motility agents. Outcomes assessed included TDCP, TDFXC, TDIEN, CPOT score, first catheter satisfaction, number of adjustments, time to first catheterization, first catheter success rate, and jejunal reach prediction. No significant differences were observed across these subgroups.

## 4 Discussion

### 4.1 Superior performance of ENG-P in placement efficiency and accuracy

The present investigation revealed that the initial success rate of ENG-P (81.36%) substantially exceeded that of the CBN-P (65.85%, *P* = 0.042). Furthermore, the procedural duration was markedly reduced in the ENG-P cohort (median 18 min vs. 20 min, *P* < 0.001), accompanied by fewer reinsertion attempts (median 0 vs. 1, *P* = 0.001). These disparities primarily stem from the real-time trajectory visualization capability of ENG-P, which enables continuous monitoring of catheter positioning. The generated three-dimensional pathway mapping effectively circumvents the risks of airway misplacement and gastric folding, thereby minimizing the necessity for repeated manipulations associated with blind advancement (38, 42). Conversely, CBN-P depends on indirect assessment methods including auscultation and pH measurement, which are significantly influenced by operator expertise. The blind insertion approach is more susceptible to failure owing to anatomical variations such as gastric hypomotility or pyloric spasm, consequently elevating procedural complexity and contributing to a higher initial failure rate (43). While the overall success rate of ENG-P in this investigation was comparatively lower than that documented in previous studies, this discrepancy may be ascribed to the sedation and analgesia typically administered to mechanically ventilated patients, which can affect gastrointestinal motility and augment procedural challenges (18, 44). After statistical calibration, the overall success rates were 87.68% for CBN-P and 96.15% for ENG-P, with no statistically significant intergroup difference. Nevertheless, the adjusted success rate aligns with findings from analogous studies (45).

Additionally, the ENG-P group exhibited a significantly enhanced success rate in jejunal placement prediction (81.36%) relative to the CBN-P group (40.24%, *P* < 0.001), accompanied by a substantially reduced “unclear” rate (5.08% vs. 41.46%). This underscores that electromagnetic navigation not only furnishes real-time procedural guidance but also augments the operator’s capacity to anticipate placement outcomes through catheter trajectory visualization. This advancement diminishes the requirement for secondary adjustments due to ambiguous positioning, thereby holding substantial clinical significance in enhancing procedural certainty in intensive care unit settings (46).

### 4.2 Value of ENG-P in medical resource optimization and economic efficiency

The investigation revealed that the ENG-P cohort exhibited significantly fewer instances of X-ray utilization [median 1(1, 1) vs. 1(1, 1.75), *P* = 0.004] and lower aggregate catheterization expenditures (median 886 CNY vs. 952 CNY, *P* = 0.005) in comparison to the CBN-P group. These outcomes are directly correlated with enhanced procedural efficacy: the superior initial success rate of ENG-P diminished the necessity for repeated X-ray confirmations due to procedural failures or ambiguous positioning. Furthermore, the trajectory visualization capability of ENG-P reduced reliance on supplementary procedures such as interventional fluoroscopy and endoscopic catheter placement. In the CBN-P group, four patients necessitated catheterization outside the intensive care unit, whereas only one patient from the ENG-P group required such intervention. This indirectly mitigated the consumption of human and equipment resources.

From a health economics standpoint, the cost advantage of ENG-P is not attributable to a reduction in single-use material costs, but rather through the diminution of “trial-and-error” expenditures. These encompass the wastage of consumables from repeated catheterizations, radiation exposure risks from multiple X-ray examinations, and the temporal investment of healthcare personnel (47, 48). Analogous to ultrasound equipment, electromagnetic navigation devices represent a one-time investment with long-term utilization potential, rendering them particularly suitable for resource-constrained intensive care unit settings. This approach offers novel insights for the efficient allocation of bedside procedural resources (29, 46, 49).

### 4.3 Timeliness of clinical decision-making: accelerating early initiation of enteral nutrition

The clinical timeliness metrics indicated no significant intergroup differences in the time from decision to catheter placement (TDCP) and the time from decision to first X-ray confirmation (TDFXC). This is likely attributable to the scheduling of catheter placements during afternoon idle periods, with X-ray imaging typically arranged for the afternoon. However, the ENG-P group demonstrated a significantly abbreviated time from decision to initiation of enteral nutrition (TDIEN) relative to the CBN-P group (9.0 h vs. 11.0 h, *P* = 0.001). This finding suggests that ENG-P does not expedite nutrition initiation by shortening the procedure itself, but rather by augmenting the initial success rate and positional certainty, thereby reducing delays attributable to failures or unclear positioning. Moreover, clinicians acknowledge that electromagnetic navigation offers accuracy comparable to X-ray, leading to the initiation of feeding in some patients even prior to X-ray confirmation (50).

Existing research and clinical consensus suggest that early enteral nutrition (within 48 h) can reduce infection-related complications and improve clinical outcomes (51, 52). While our study did not directly measure infection rates, ENG-P facilitated faster initiation of enteral nutrition by reducing TDIEN. The visualization navigation enabled immediate positional confirmation, thus avoiding repeated X-rays and adjustments and allowing for prompt feeding commencement. In contrast, the CBN-P group had higher initial failure rates, and some patients experienced a cycle of “placement—failure—readjustment—confirmation,” which indirectly delayed the initiation of nutritional support (49, 53).

### 4.4 Safety and overall efficacy: non-inferiority and potential advantages of ENG-P

Although no significant intergroup differences were observed in CPOT scores (2 vs. 3, *P* = 0.074) and overall catheter placement success rates (84.75% vs. 78.05%, *P* = 0.253), the ENG-P group exhibited lower pain scores and a higher overall success rate. The incidence of adverse events, such as mucosal injury and coughing, was marginally lower in the ENG-P group (mucosal injury: 5.08% vs. 8.54%; coughing: 8.47% vs. 9.76%), with no severe malposition events necessitating emergency intervention. This indicates that while ENG-P enhances procedural efficiency, it does not elevate operational risks, maintaining safety comparable to traditional methods.

It is noteworthy that the overall success rate of the CBN-P group improved through multiple blind attempts, which could exacerbate patient discomfort, such as repeated mucosal irritation and the overall healthcare burden. Due to the absence of trajectory visualization, blind attempts are prone to airway misplacement into the thoracic cavity (31). In contrast, ENG-P offers a straightforward, easily mastered procedure with a high initial success rate. This aligns with the intensive care unit’s transition toward minimally invasive and patient-comfort-oriented procedures, particularly for mechanically ventilated patients who cannot tolerate repeated operations (54).

## 5 Limitations and future directions

This investigation is subject to several limitations: (1) The non-randomized, prospective cohort design may introduce selection bias. The collection of pertinent metrics in the intensive care unit setting is challenging without double-blinding, and the progressive enhancement of operator proficiency over time may have accentuated the advantages of ENG-P. (2) The sample size was limited to 146 patients, and the study excluded individuals with gastrointestinal abnormalities, awake patients, and those in the prone position, which may restrict the generalizability of the findings. In addition, the relatively small sample size may have limited the statistical power to detect differences in secondary outcomes. Although subgroup analyses were performed, the study may still be underpowered to identify subtle differences among subgroups. (3) The institution lacks a standardized fee structure for electromagnetic navigation, and the cost calculations did not incorporate the amortized one-time purchase cost of the device over multiple uses, which may result in an underestimation of the true expenses.

Future research endeavors can address these limitations through several avenues: (1) Conduct multicenter randomized controlled trials to validate the efficacy, cost-effectiveness, and timeliness of ENG-P across diverse intensive care unit populations. (2) Explore technological advancements, such as the integration of visual artificial intelligence path prediction and real-time positional algorithms, to enhance catheter placement accuracy in complex anatomical scenarios. (3) Transition from reusable electromagnetic navigation sensing catheters to single-use variants to mitigate the risks of bacterial colonization and infection associated with repeated sterilization. (4) Integrate electromagnetic path visualization with endoscopic local visualization to establish a dual-path and local visualization guidance system, thereby further augmenting the safety and efficiency of nasoenteric tube placement and providing more robust evidence for the clinical adoption of ENG-P.

## 6 Conclusion

ENG-P outperforms CBN-P in terms of first-attempt success rate, procedural efficiency, and medical economics, while maintaining similar safety standards. It offers significant benefits in mechanically ventilated patients, including reduced procedure time, fewer reintubations, and earlier enteral nutrition initiation. Consequently, ENG-P is the optimal technique for bedside NJT placement in ICU settings (55).

## Data Availability

The original contributions presented in the study are included in the article/supplementary material, further inquiries can be directed to the corresponding author.

## Footnotes

1
http://www.medsta.cn/software
